# Left Ventricular Pseudoaneurysm After Sandwich Patch Repair via Right Ventriculotomy for Postinfarction Ventricular Septal Perforation

**DOI:** 10.1016/j.atssr.2025.02.013

**Published:** 2025-03-13

**Authors:** Satoru Tomita, Naonori Kawamoto, Satoshi Kainuma, Kota Suzuki, Takashi Kakuta, Masaya Hirayama, Satsuki Fukushima

**Affiliations:** 1 Department of Cardiovascular Surgery, National Cerebral and Cardiovascular Center, Osaka, Japan

## Abstract

This report describes 2 rare cases of left ventricular pseudoaneurysm (LVPA) after sandwich patch repair through a right ventriculotomy for postinfarction ventricular septal perforation. The first case involved an 80-year-old man who experienced LVPA 8 months after undergoing the procedure. The second case involved a 63-year-old man with LVPA that was diagnosed incidentally 3 years postoperatively. In both cases, a large tear was observed along the previous anterior left ventricular wall suture line, and it indicated insufficient infarct exclusion at the patch repair site. Each patient successfully underwent patch repair.

Ventricular septal perforation (VSP) after acute myocardial infarction (AMI) leads to rapid hemodynamic deterioration, resulting in high surgical mortality rates in patients undergoing post-AMI VSP repair.^1^ The extended sandwich patch technique through a right ventriculotomy is effective for improving early survival and preventing residual shunt recurrence.^2^ However, the right ventricular approach involves the complex passage of suture needles through the left ventricle, thus making complete exclusion of the anterior left ventricular (LV) wall infarct area challenging.^3^ Additionally, the long-term consequences of incomplete infarct exclusion after VSP repair through a right ventriculotomy remain unclear. This report presents 2 cases of LV pseudoaneurysm originating from unexcluded anterior infarcted myocardium after sandwich patch repair through a right ventriculotomy.

## Case Reports

### Patient 1

**Figure 1:**
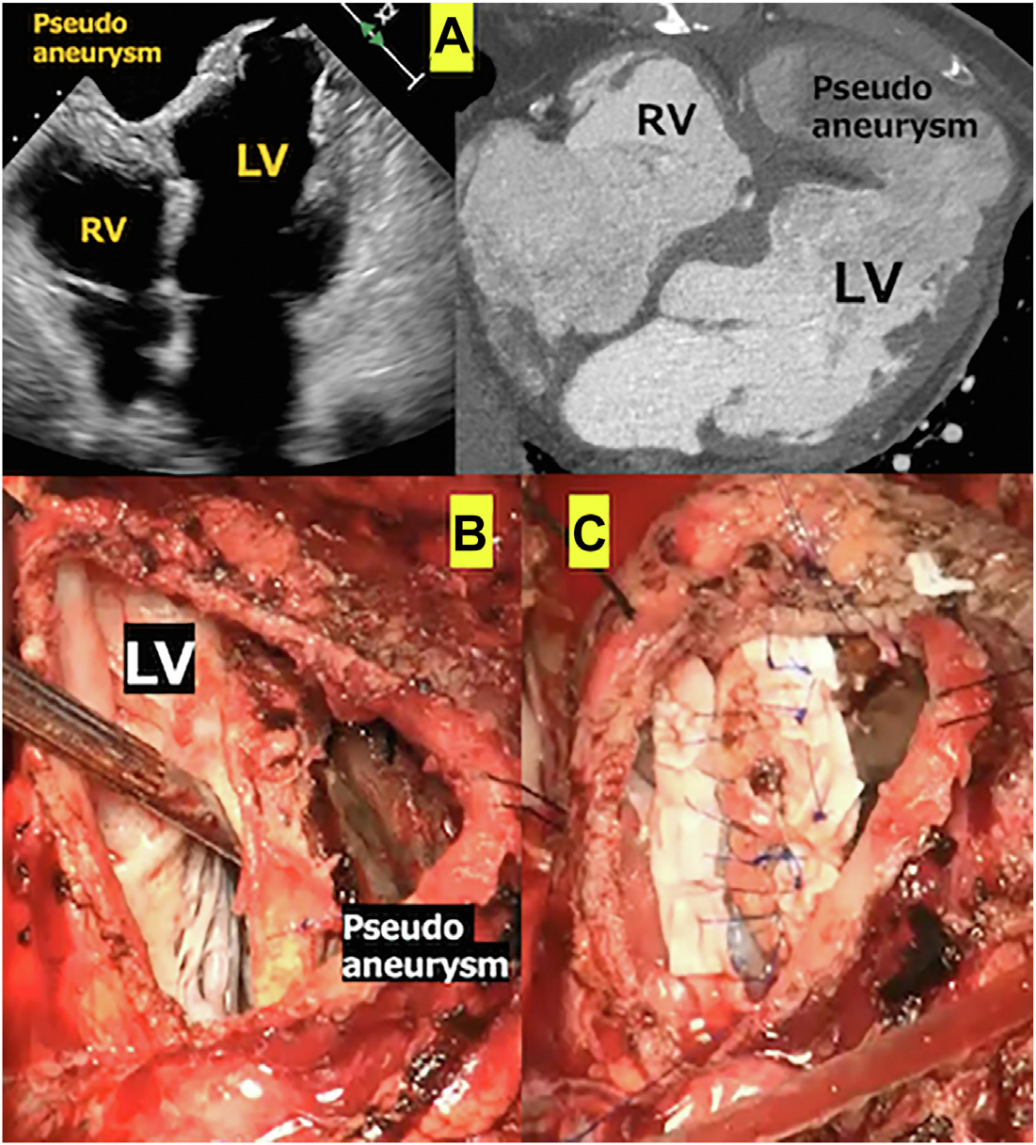
(A) Echocardiographic and contrast-enhanced computed tomographic findings. Pseudoaneurysm formation was noted in the left ventricular apex. (B) Left ventricular side observation after left ventricular apex pseudoaneurysm excision. Left and right ventricular patches were joined without a shunt, thus suggesting thinning of the left ventricular infarct myocardium outside of the septal patch as the causative factor. (C) Surgery was performed to resect the apex pseudoaneurysm wall and conduct left ventriculoplasty with a bovine pericardial patch on the left ventricular side. (LV, left ventricle; RV, right ventricle.)

An 80-year-old man underwent a sandwich patch repair through a right ventricular approach^4^ for VSP in the ventricular apex 1 week after AMI onset. Postoperative echocardiography showed no evidence of a residual shunt, and the patient was discharged on postoperative day 39. Eight months later, a chest roentgenogram revealed cardiac silhouette enlargement. Subsequent echocardiography and contrast computed tomography confirmed a large pseudoaneurysm in the LV apex (Figure 1A). Although the patient’s condition remained stable, immediate surgery was planned.

**Figure 2:**
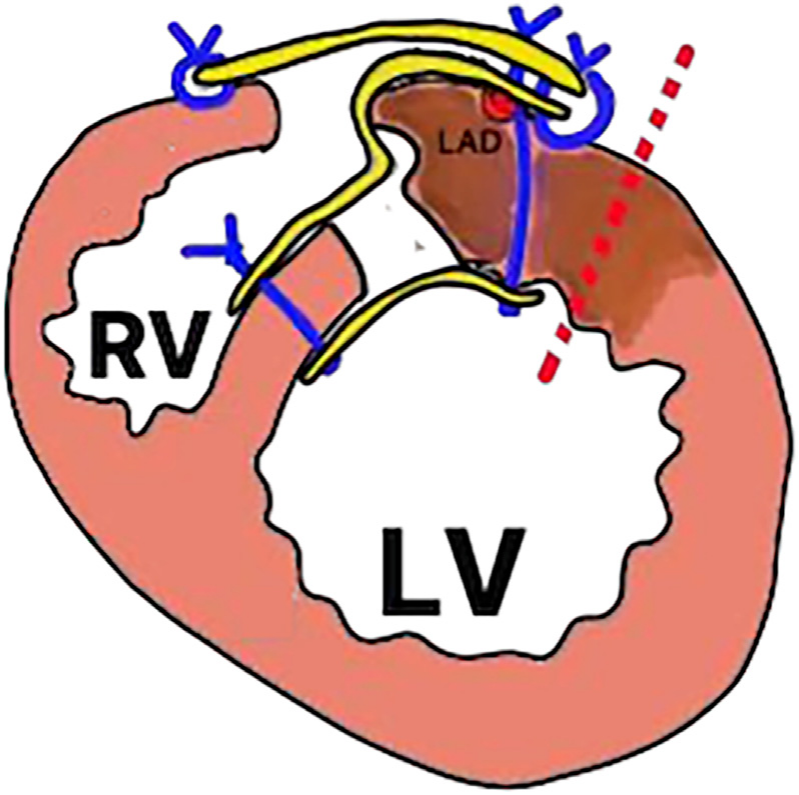
Pseudoaneurysm originating from the left ventricular infarcted myocardium along the septal patch suture line (blue strings, previous sutures; red dotted line, tear; yellow lines, previous ventricular septal perforation repair patches). (LAD, left anterior descending artery; LV, left ventricle; RV, right ventricle.)

Before a median repeat sternotomy, cardiopulmonary bypass (CPB) was established using the right-sided femoral artery, femoral vein, and internal jugular vein. Adhesions from the anterior surface of the right ventricle to the lateral wall of the left ventricle were noted and dissected under cardiac arrest. On opening the LV apex pseudoaneurysm, a large defect was observed extending from the anterior LV wall to the apex near the previous patch suture line (Figure 1B). This finding suggested that the defect had developed from the infarcted LV myocardium along the suture line (Figure 2). A bovine pericardial patch was secured with a 3-0 polypropylene (Prolene, Ethicon) running suture around the neck of the defect. The pseudoaneurysm wall was closed over the patch using a 4-0 Prolene mattress suture with 2 polytetrafluoroethylene (Teflon, Chemours) felt patches reinforcing the suture line (Figure 1C). The cross-clamp, CPB, and total operative times were 65, 167, and 359 minutes, respectively. Postoperative echocardiography confirmed the absence of an intraventricular shunt or apex aneurysm recurrence, and the patient was discharged 75 days later.

### Patient 2

**Figure 3:**
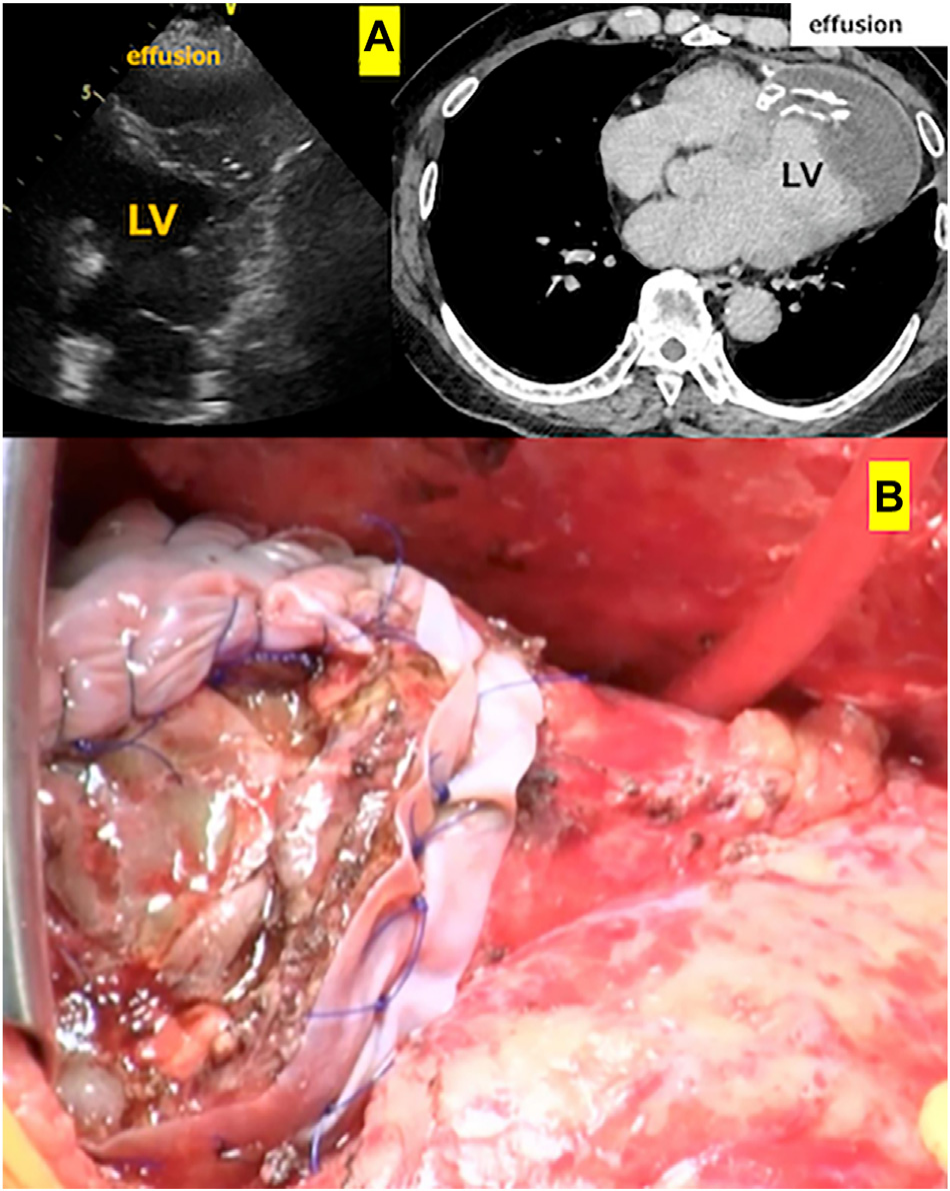
(A) Preoperative echocardiogram and contrast-enhanced computed tomographic findings showed effusion around the left ventricular apex. (B) Surgery was performed to repair pseudoaneurysm with bovine pericardial patch. (LV, left ventricle.)

A 63-year-old man with AMI underwent LV repair with a bovine pericardial patch for LV free wall rupture (anterior wall) and sandwich patch repair through a right ventricular approach. Postoperative echocardiography showed no residual shunt. Three years later, the patient experienced dyspnea and fever. A chest roentgenogram revealed cardiac enlargement, and echocardiography and contrast computed tomography confirmed a massive effusion around the heart apex (Figure 3A). Suspecting a hematoma or abscess, immediate drainage was performed.

Surgery was performed using general anesthesia with the patient in the supine position. During a right thoracotomy, fresh blood gushed out immediately on opening the apex pericardium, thereby confirming pseudoaneurysm rupture. Conversion to a full sternotomy became necessary. CPB was established using the left femoral artery and right femoral vein. Adhesions around the previous lateral LV wall and apex patches were dissected. At the apex, inflammation had caused thickening and sclerosis of the epicardium, with a large tear observed in the LV anterior wall between the LV patch and the suture line of the right ventricular patch from the previous VSP repair. Pseudoaneurysm repair was performed using a bovine pericardial patch, as in patient 1 (Figure 3B). The cross-clamp, CPB, and operative times were 57, 174, and 354 minutes, respectively. The patient’s postoperative course was uneventful, with no intraventricular shunt or apical aneurysm recurrence, and he was discharged 69 days postoperatively.

## Comment

Infarct exclusion by suturing a bovine pericardial patch to healthy endocardium through a left ventriculotomy is effective because it directly excludes the LV infarct area.^5^ However, VSP repair through a left ventriculotomy carries a high risk of mortality.^2^ The extended sandwich patch technique through a right ventriculotomy has been shown to improve early survival and reduce residual shunt formation.^2^ Despite this, whether the patch can achieve complete exclusion of the infarcted myocardium remains uncertain. This uncertainty arises because sandwich patch repair through a right ventriculotomy is performed in a nearly blind manner, with the LV patch positioned by passing a needle through a small septal defect rather than directly opening the left ventricle to place the patch. Consequently, this technique may not provide sufficient exclusion of the infarct area.

Pseudoaneurysms arising from the suture line after postinfarction VSP repair through an LV approach are documented.^3^^,^^6^, ^7^, ^8^ However, late pseudoaneurysms after sandwich repair through a right ventriculotomy without a left ventriculotomy are rare.^3^ Previous studies of pseudoaneurysms after VSP repair through a left ventriculotomy have identified technical failure and suture breakdown from excessive tension in fragile myocardium after myocardial infarction as causes of ventriculotomy closure line pseudoaneurysms. Although LV patches should be sewn to healthy myocardium beyond the infarction, determining the appropriate site or evaluating the suture-holding strength of the surrounding myocardium is challenging.^3^ In our patients, LV apex tears occurred along the sandwich patch suture line, likely from insufficient infarct exclusion, leading to suture breakdown as a result of intolerable tension in the fragile infarcted myocardium. By May 2024, our center (National Cerebral and Cardiovascular Center, Osaka, Japan) had performed 26 sandwich patch repairs through a right ventriculotomy, with these pseudoaneurysm cases occurring within several months. Although pseudoaneurysms may occur more frequently after right than left ventriculotomy repairs, the former provides better survival and fewer residual shunts. Adequately sizing the sandwich patch to fully exclude the infarcted myocardium is essential. Further studies and case series are needed to clarify the long-term effects of sandwich patch repair through a right ventriculotomy.

In conclusion, we have presented 2 rare cases of LV pseudoaneurysm after sandwich patch repair through a right ventriculotomy, both of which were successfully managed with surgical repair.
